# Calgary Preschool magnetic resonance imaging (MRI) dataset

**DOI:** 10.1016/j.dib.2020.105224

**Published:** 2020-01-31

**Authors:** Jess E. Reynolds, Xiangyu Long, Dmitrii Paniukov, Mercedes Bagshawe, Catherine Lebel

**Affiliations:** a Department of Radiology, University of Calgary, 2500 University Drive NW, Calgary, T2N 1N4, AB, Canada; b Child and Adolescent Imaging Research Program, University of Calgary, Calgary, Canada; c Alberta Children's Hospital Research Institute, University of Calgary, Calgary, Canada; d Owerko Centre, Alberta Children Hospital Research Institute, University of Calgary, 2500 University Drive NW, Calgary, AB, T2N 1N4, Canada; e Hotchkiss Brain Institute, University of Calgary, 2500 University Drive NW, Calgary, AB, T2N 1N4, Canada; f Department of Pediatrics, University of Calgary, 2500 University Drive NW, Calgary, AB, T2N 1N4, Canada

**Keywords:** Magnetic resonance imaging, Arterial spin labeling, Cerebral blood flow, Diffusion tensor imaging, Resting state fMRI, Passive viewing fMRI, Neurodevelopment, Early childhood

## Abstract

The Calgary Preschool MRI Dataset in the Developmental Neuroimaging Lab at the University of Calgary uses magnetic resonance imaging (MRI) techniques to study brain structure and function in early childhood [1–3]. The dataset aims to characterise brain development in early childhood (2–8 years), and to understand links to cognitive and behavioral development, as well as provide a baseline from which to identify atypical development in children with diseases, disorders, or brain injuries. MRI data are provided for 126 children (61 males, 65 females). Children ranged from 1.95 to 6.22 years (mean = 3.98 ± 1.06 years) at the time of their first scan and were initially scanned at six month intervals, and now continue to be followed annually (1–20 scans per child, 431 total datasets; datasets do not always have all scan modalities available). All MRI scans were acquired on the same General Electric 3T MR750w system and 32-channel head coil (GE, Waukesha, WI) at the Alberta Children's Hospital in Calgary, Canada. The MRI protocols provided in this dataset include: T1-weighted images acquired using a FSPGR BRAVO sequence; arterial spin labeling (ASL) images acquired with the vendor supplied pseudo continuous 3D ASL sequence; diffusion tensor imaging data acquired using single shot spin echo echo-planar imaging; and passive viewing resting state functional MRI data acquired with a gradient-echo echo-planar imaging sequence.

**Table:** Specifications Table

Subject	Developmental Neuroscience
Specific subject area	Magnetic Resonance Imaging in Early Childhood; Brain development
Type of data	NIfTI image files (MRI) T1-weighted Imaging Dataset Arterial Spin Labelling (ASL) Dataset Diffusion Tensor Imaging (b = 750 s/mm^2^) Dataset Passive viewing functional MRI Dataset Accompanying Microsoft Office Excel Spreadsheet
How data were acquired	Magnetic Resonance Imaging Data were acquired using the same General Electric 3T MR750w system and 32-channel head coil (GE, Waukesha, WI) at the Alberta Children's Hospital in Calgary, Canada
Data format	Raw (quality checked)
Parameters for data collection	T1-weighted images were acquired using a FSPGR BRAVO sequence. ASL images were acquired with the vendor supplied (GE) pseudo continuous 3D ASL sequence. Diffusion tensor imaging data were acquired using a single shot spin echo echo-planar imaging sequence. Passive viewing fMRI data were acquired with a gradient-echo echo-planar imaging (EPI) sequence.
Description of data collection	Children were recruited from the local community and an ongoing prospective study (APrON) in Calgary, Canada [4]. All imaging was conducted using the same General Electric 3T MR750w system and a 32-channel head coil (GE, Waukesha, WI) at the Alberta Children's Hospital in Calgary, Canada. Children were scanned without sedation, either while they were awake and watching a movie of their choice, or while they were sleeping.
Data source location	University of Calgary Calgary/Alberta Canada
Data accessibility	Public Repository Open Science Framework DOI 10.17605/OSF.IO/AXZ5R https://osf.io/axz5r/
Related research article	Dmitrii Paniukov, R Marc Lebel, Gerald Giesbrecht, Catherine Lebel Cerebral blood flow increases across early childhood NeuroImage https://doi.org/10.1016/j.neuroimage.2019.116224

**Table:** 

**Value of the Data** • These data provide longitudinal MRI scans across early childhood (2–8 years), an age range that has received limited research attention, in part due to the difficulties associated with scanning young children (e.g., motion). Limited MRI data is currently available for researchers to access in this age range, making this dataset a valuable resource. • These data will be beneficial for anyone interested in researching typical brain development in early childhood using MRI • These data provided can be analysed and/or compared with other datasets, or used as pilot data for the development of future research projects. They can be used to detail structural and/or functional brain development, or as baseline data to help to identify deviations from typical development in children with various diseases, disorders, or brain injuries.

## 1 Data

Four types of images are currently provided on OSF as part of the ‘Calgary Preschool MRI Dataset’ [5]: 1) T1-weighted anatomical imaging; 2) arterial spin labeling (ASL) cerebral blood flow (CBF) maps; 3) diffusion tensor imaging (DTI); and 4) passive viewing (resting state) fMRI. These four modalities were all part of the same protocol, but not all children completed all imaging sequences (due to time), while some sequences were motion-corrupted and removed; thus, not all children have data available for all four modalities. All of the NIfTI files provided were converted from raw DICOM images using dcm2nii from the mricron software package [6]. An accompanying excel sheet ‘Calgary_Preschool_Dataset’, appended with the date of last data update, provides details on age, sex, and longitudinal participant data (Calgary_Preschool_Dataset_Updated_20200116.xlsx at time of publication). This spreadsheet also contains pre-reading assessment information and maternal education (years postsecondary). Data collection is ongoing and new files are uploaded periodically. Participant IDs are unique for each individual, while scan IDs are unique for each scan session. These can therefore be used to match data from the same participant (e.g., longitudinal data acquired at different times) or different modalities acquired in the same session (e.g., DTI and ASL).

For the T1 data (‘T1_dataset’ folder), a folder for each participant contains subfolders for each scan time point, which contains the T1-weighted NIfTI file. For the ASL data (‘ASL_dataset’ folder), a folder for each participant contains subfolders for each scan time point. Within the timepoint subfolder, the ‘anat’ subfolder contains the raw T1-weighted anatomical scan, and the ‘asl’ subfolder contains the CBF map. For both the DTI (‘DTI_Dataset_b750’) and passive viewing fMRI (‘REST_dataset’), folders labeled by participant ID contain NIfTI files for each scan time point. Within the ‘DTI_Dataset_b750’ folder, a subfolder named ‘Calgary_Preschool_DTI_b750_Dataset_Information’ contains a the bval, bvec, and bmatrix files for the dataset; these were identical for all individuals.

## 2 Experimental design, materials, and methods

All imaging was conducted by the Developmental Neuroimaging Lab at the University of Calgary (https://www.developmentalneuroimaginglab.ca) using the same General Electric 3T MR750w system and a 32-channel head coil (GE, Waukesha, WI) at the Alberta Children's Hospital in Calgary, Canada. Children were scanned without sedation, either while they were awake and watching a movie of their choice, or while they were sleeping. T1-weighted images, ASL, DTI, and passive viewing fMRI data were acquired as part of a longer MRI protocol (∼45–60 minutes total). Parents were provided with information on MRI procedures before the appointment, and were given the option to complete a practice MRI session in a training scanner [7]. A book about the MRI experience at the Alberta Children's Hospital was written specifically for this data collection and provided to families before their scan (it is freely available as an e-book: http://www.lulu.com/shop/ashleigh-frayne/pluto-and-the-mri-rocket-ship-adventure/ebook/product-22122518.html).

Children were recruited from the local community and from an ongoing prospective study in Alberta, Canada [4]; all children lived in the Calgary area. MRI data are provided for 126 children (61 males, 65 females). Children ranged from 1.95 to 6.22 years (mean = 3.98 ± 1.06 years) at the time of their first scan and were initially invited to return at six month intervals; many continue to be followed annually (1–20 scans per child, 431 total datasets; children do not always have all scan modalities available). All children were free from genetic disorders and significant intellectual or motor impairments and were born ≥35 weeks' gestation. Parental/guardian written informed consent, and child assent were obtained for each participant. The University of Calgary Conjoint Health Research Ethics Board (CHREB) approved this research project (REB13-0020).

In addition to the imaging data, on the same day as scanning, children over 3 years had their pre-reading skills assessed using the NEPSY-II Phonological Processing and Speeded Naming subtests (approx. 20 mins). The Phonological Processing subtest assesses phonemic awareness and the Speeded Naming subtest assesses rapid semantic access to, and production of, names of colors and shapes (Korkman et al., 2007). Where currently available raw, and age standardized scores are provided for these two NEPSY-II subtests in the Calgary_Preschool_Dataset' excel sheet.

New data are periodically uploaded to the dataset; the accompanying excel sheet is updated to reflect additions (updated date appended to the filename) and has a column for the scan upload date.

### 2.1 MRI scan acquisition parameters and quality assurance.

#### 2.1.1 T1-weighted

T1-weighted images were acquired using a FSPGR BRAVO sequence: 0.9 × 0.9 × 0.9mm resolution (resampled on the scanner 0.45 × 0.45 × 0.9mm), 210 axial slices, TR = 8.23 ms, TE = 3.76 ms, flip angle = 12°, matrix size = 512 × 512, inversion time = 540 ms. Total imaging time was 4:26 min:sec. All uploaded T1-weighted scans passed quality checked for motion corruption and scanner artefacts.

#### 2.1.2 Arterial spin labeling

ASL images were acquired with the GE vendor supplied pseudo continuous 3D ASL sequence: 3.5 × 3.5 × 4 mm resolution (interpolated on scanner resolution of 1.73 × 1.73 × 4 mm), 30 axial slices, TR = 4.56 s, TE = 10.74 ms, post label delay of 1.5 seconds (in accordance to a recent consensus paper [8]). The label plane was positioned 2.2 cm below the base of the imaging volume, which was placed at the base of the cerebellum in all participants. The sequence scan time was 3:11 min:sec.

ASL perfusion DICOM images were converted to CBF DICOM images on the scanner computer using the vendor supplied approach (consistent with [8]). We used the vendor standard M0 image for quantification. This was a 2 second saturation recovery image. We performed quantification using the general kinetic model proposed by Buxton and colleagues [9].

CBF datasets underwent a visual quality check (DP), and datasets with excessive motion that produced sharp ringing artefacts and/or blurred brain structures on T1 and/or CBF, as well as datasets with scanner artefacts were excluded. T1 data is provided alongside the ASL data for ease of analysis; this data is identical to that provided in the “T1_dataset” folder.

Only scans that passed quality assurance have been provided as part of this dataset. For all of these scans, children were awake at the end of the scanning protocol, so all children are presumed to have been awake for the ASL sequence.

#### 2.1.3 Diffusion tensor imaging

Diffusion tensor imaging (DTI) data were acquired using single a shot spin echo echo-planar imaging sequence: 1.6 × 1.6 × 2.2 mm resolution (resampled on scanner to 0.78 × 0.78 × 2.2 mm), full brain coverage, FOV = 20.0, TR = 6750 ms; TE = 79 ms (set to minimum for first year of data collection), 30 gradient encoding directions at b = 750 s/mm^2^, and five interleaved images without gradient encoding at b = 0 s/mm^2^ for a total acquisition time of 4:03 min:sec.

Raw NIfTI datasets were quality checked for motion corruption and scanner artefacts to ensure all data had a minimum of two high quality b = 0 s/mm^2^ volumes, and 18 high quality diffusion weighted volumes. We removed motion-corrupted volumes for analysis, but raw data, including volumes that are motion corrupted, have been provided online.

#### 2.1.4 Passive viewing fMRI

Passive viewing fMRI data were acquired with a gradient-echo echo-planar imaging (EPI) sequence, 3.59 × 3.59 × 3.6 mm resolution, 36 axial slices, TR = 2000 ms, TE = 30 ms, flip angle = 60°, matrix size = 64 × 64, 250 vol, for a total acquisition time of 8:10 min:sec. Children were watching a movie of their choice during this scan (movie choice was not recorded).

Following slice timing and head motion detection, the averaged relative frame-wise displacement (FD) was calculated using FSL [10]. A spike matrix was created from volumes that had high relative FD (>0.3 mm) [11]. Datasets with spike volumes longer than 4 minutes (i.e., less than 4 minutes of low-motion data remaining) were excluded from the raw dataset. Only fMRI scans during which the child was awake have been included in this dataset.

The following publications used the T1 [12,13], ASL [2], DTI [3,12,[14], [15], [16], [17]], and passive viewing fMRI [1,13,16] imaging datasets to characterise brain development and its links with cognition and behaviour across early childhood.

## CRediT author statement

**J.E.R** Conceptualization, Methodology, Formal analysis, Data Curation, Writing - Original Draft, Writing - Review & Editing, Visualization **X.L.** Conceptualization, Methodology, Software, Formal analysis, Data Curation, Writing - Original Draft, Writing - Review & Editing, Visualization **D.P.** Conceptualization, Methodology, Software, Formal analysis, Data Curation, Writing - Review & Editing, Visualization **M.B.** Methodology, Formal analysis, Investigation, Data Curation, Writing - Review & Editing, Visualization, Project administration **C.L.** Conceptualization, Methodology, Formal analysis, Investigation, Writing - Review & Editing, Supervision, Project administration, Funding acquisition.
